# CDK9 Expression Shows Role as a Potential Prognostic Biomarker in Breast Cancer Patients Who Fail to Achieve Pathologic Complete Response after Neoadjuvant Chemotherapy

**DOI:** 10.1155/2018/6945129

**Published:** 2018-10-14

**Authors:** Ashley J. Schlafstein, Allison E. Withers, Soumon Rudra, Diana Danelia, Jeffrey M. Switchenko, Donna Mister, Saul Harari, Hui Zhang, Waaqo Daddacha, Shahrzad Ehdaivand, Xiaoxian Li, Mylin A. Torres, David S. Yu

**Affiliations:** ^1^Department of Radiation Oncology, Winship Cancer Institute, Emory University School of Medicine, Atlanta, GA 30322, USA; ^2^Department of Biostatistics and Bioinformatics, Rollins School of Public Health and Winship Cancer Institute, Emory University School of Medicine, Atlanta, GA 30322, USA; ^3^Department of Pathology, Winship Cancer Institute, Emory University School of Medicine, Atlanta, GA 30322, USA

## Abstract

Failure to achieve pathologic complete response is associated with poor prognosis in breast cancer patients following neoadjuvant chemotherapy (NACT). However, prognostic biomarkers for clinical outcome are unclear in this patient population. Cyclin-dependent kinase 9 (CDK9) is often dysregulated in breast cancer, and its deficiency results in genomic instability. We reviewed the records of 84 breast cancer patients from Emory University's Winship Cancer Institute who had undergone surgical resection after NACT and had tissue available for tissue microarray analysis (TMA). Data recorded included disease presentation, treatment, pathologic response, overall survival (OS), locoregional recurrence free survival (LRRFS), distant-failure free survival (DFFS), recurrence-free survival (RFS), and event-free survival (EFS). Immunohistochemistry was performed on patient samples to determine CDK9 expression levels after NACT. Protein expression was linked with clinical data to determine significance. In a Cox proportional hazards model, using a time-dependent covariate to evaluate the risk of death between groups beyond 3 years, high CDK9 expression was significantly associated with an increase in OS (HR: 0.26, 95% CI: 0.07-0.98, p=0.046). However, Kaplan-Meier curves for OS, LRRFS, DFFS, RFS, and EFS did not reach statistical significance. The results of this study indicate that CDK9 may have a potential role as a prognostic biomarker in patients with breast cancer following NACT. However, further validation studies with increased sample sizes are needed to help elucidate the prognostic role for CDK9 in the management of these patients.

## 1. Introduction

Patients with breast cancer with high-risk features, such as triple-negative breast cancers (TNBC), are often treated with neoadjuvant chemotherapy (NACT) prior to surgical resection [1]. NACT can reduce the size of the breast tumor, subsequently allowing for breast-conservation surgery in patients who otherwise might require a complete mastectomy due to large tumor size [2]. In addition, survival outcomes are significantly impacted by response to NACT. Patients with residual invasive breast cancer following NACT are reported to have a high risk of relapse [3], yet, some of these patients achieve long-term survival without recurrence. Thus, there is a need to identify biomarkers for patients with residual disease following NACT that can provide both prognostic information regarding outcomes and predictive information to identify patients with more aggressive disease who could benefit from additional therapies.

Cyclin-dependent kinase 9 (CDK9) is a component of the positive transcription elongation factor (P-TEFb) complex, which phosphorylates the carboxyl-terminal domain (CTD) of RNA polymerase II to promote transcription elongation [4]. We and others have also reported that CDK9 has a transcription-independent function in promoting genome integrity [5–11]. CDK9 facilitates recovery from replication stress and interacts with ATR and several cell cycle checkpoint proteins. Furthermore, its loss results in spontaneous replication stress and genetic instability [10]. CDK9 also promotes DNA double-strand break (DSB) repair by homologous recombination (HR) by facilitating recruitment of the BRCA1 breast tumor suppressor protein to DNA damage sites [6] and associates with Ku70, which is involved in DSB repair by non-homologous end joining (NHEJ) [5]. CDK9's role in promoting genome integrity is regulated at least in part through deacetylation by the SIRT2 sirtuin deacetylase and breast tumor suppressor protein, which promotes its kinase activity [11] and in turn phosphorylates UBE2A and directs H2Bub1 and PCNA ubiquitinylation [12].

CDK9 is a protein of potential clinical significance in oncology because of its role in promoting genomic integrity in response to agents that induce replication stress and DNA damage. It has been shown to be dysregulated in a number of malignancies, including breast cancer [13, 14]. CDK9 inhibitors have also been shown to inhibit growth of breast cancer cells and tumors [15–18]. In this study, we evaluated CDK9 expression in post-NACT breast tumors.

## 2. Materials and Methods

### 2.1. Patient Selection

The patient cohort was selected from a prospectively maintained, IRB approved database of patients who underwent surgical resection for breast cancer between 2001 and 2011 at Emory University's Winship Cancer Institute. A total of 84 patients who received NACT prior to surgical resection without achieving complete pathological response had tissue from their surgical resections available for analysis. These patients were selected for inclusion in the study. Patient demographics, pathologic information, and treatment outcomes were obtained from electronic medical records. Staging was based on the AJCC 7th edition. Complete pathological response was defined as no invasive cancer in the breast or axilla. Permission for patient information used in this study was obtained from Emory's Institutional Review Board and patient confidentiality was maintained in accordance with the Health Insurance Portability and Accountability Act of 1996.

### 2.2. Tissue Microarray (TMA) Development

TMAs were used for this study in order to analyze tissues from multiple patients on a single slide and thus reduce the time and cost for analysis. In order to construct the TMAs, formalin-fixed paraffin embedded (FFPE) tissue blocks for each patient were acquired. These blocks contained tumor specimens from each patient's primary surgical resection. A board-certified pathologist reviewed hematoxylin and eosin (H&E) sections from each FFPE tissue block to identify representative areas of invasive ductal or lobular carcinoma. The identified area on the H&E slide was then used to guide the removal of the corresponding core from the FFPE tissue block. The removed core from each block was then placed into the TMA block in a specific row and column. Row and column location for each core was carefully recorded to ensure staining data and clinical data would be appropriately linked. Two cores of normal tissue were used as controls in every TMA constructed. These cores of normal tissue were used to assess efficacy of staining as well as to allow for comparison between cores on the same or separate TMAs for quality assurance. For more detailed information on TMA construction, please refer to the article by Rimm et al. [19].

### 2.3. TMA Staining

After the TMAs were constructed, they were cut into sections for staining. A rabbit polyclonal CDK9 antibody from Abcam at 1:1600 dilution was used in the immunohistochemistry (IHC) staining process. The TMA sections underwent antigen retrieval using either the Target Retrieval Solution (TRS) or Trilogy system. Tissue was blocked with 3% hydrogen peroxide for 5 minutes and then incubated with the primary antibody for 40 minutes. Dako's EnVision+ Dual Link System-horseradish peroxidase (HRP) was used to detect antibody-antigen interaction.

### 2.4. IHC Scoring

The TMA sections were analyzed by a board-certified pathologist who was blinded to patient outcomes. The TMA slides contained samples from each of the 84 patients in our study. Each core sample was analyzed for intensity and extent of staining. A previously validated scoring system was used to combine staining intensity (scored from 1 to 3) and extent (1-100%) into an IHC score [20]. The staining extent was then converted into a number between 1 and 3, with 1=0-50%, 2=51-80%, and 3=81-100%. IHC was then calculated according to the following formula: IHC= [(1+intensity)/3] x extent. High expression of CDK9 was defined as IHC score greater than 3, while low expression was defined as IHC score less than or equal to 3.

### 2.5. Statistics

The primary aim of this study was to determine if there was any difference in overall survival (OS) between high tumor CDK9 expression and low tumor CDK9 expression in residual tumor following NACT. Secondary outcomes analyzed included locoregional recurrence-free survival (LRFS), distant failure-free survival (DFFS), recurrence-free survival (RFS), and event-free survival (EFS). OS was calculated from date of surgery to patient death. Locoregional recurrence was defined as a biopsy proven recurrence of the primary breast cancer within the ipsilateral breast or chest wall, or in the axillary, internal mammary or supraclavicular lymph nodes. Distant failure was defined as a biopsy proven recurrence in any other location. RFS was calculated as time from date of surgery to first recurrence. Events were defined as any type of recurrence or patient death. EFS was calculated as time from date of surgery to earliest event. Survival was estimated using the Kaplan-Meier method, and log-rank p-values were reported for survival curves. Univariate cox proportional hazards models were fit for OS, LRFS, DFFS, RFS, and EFS. Additionally, time-dependent coefficient models were explored in cases where the hazards were not proportional over time. Chi-squared or Fisher's Exact tests were used to compare pathologic response across expression groups, as well as CDK9 expression across subtype and receptor groups. Complete pathologic response was defined as no residual tumor in the breast and nodes following neoadjuvant chemotherapy.

## 3. Results

A total of 84 patients were analyzed for tumor expression levels of CDK9 using the TMA staining protocol. In this cohort, 6 patients (7.1%) demonstrated a local recurrence while 23 patients (27.4%) demonstrated a distant recurrence (Table 1). Additional descriptive information including patient survival is available in Table 1. Of the 84 patients in the cohort, 67 patients' samples were analyzed for CDK9 expression. The excluded samples were not analyzed because they did not remain intact during the staining process and therefore were not able to be interpreted by the pathologist. CDK9 staining revealed that 36 patients (53.7%) had high expressing tumors and were thus classified as CDK9 positive while 31 patients (46.3%) had low expressing tumors and were classified as CDK9 negative. The median follow-up time was 8.2 years, and the range was 0.6 - 12.4 years.

**Figure 1:**
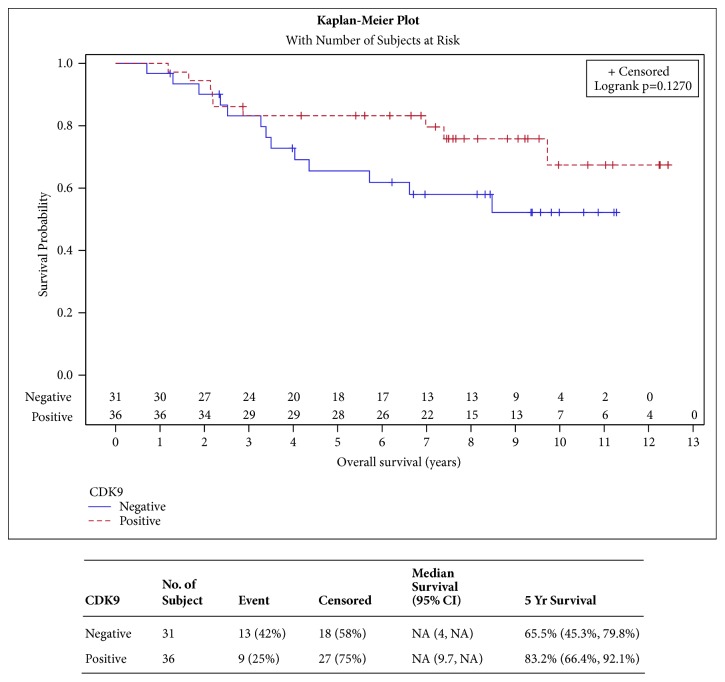
**Overall survival**. Figure 1 depicts the overall survival curves for patients with CDK9 positive versus CDK9 negative tumors. This shows a statistically nonsignificant difference in survival. However, there is a trend for increased overall survival in the group of patients with positive CDK9.

High CDK9 expression was associated with increased OS starting from the third year after the patient's initial surgery. A time-dependent covariate was fit to a Cox proportional hazards model in order to evaluate the risk of death between groups beyond 3 years, which yielded a statistically significant hazard ratio (HR): 0.26, 95% CI: 0.07-0.98, p=0.046 shown in Table 2. However, Kaplan-Meier analysis for OS did not demonstrate significant improvement with high CDK9 levels (Figure 1, p=0.127).

**Figure 2:**
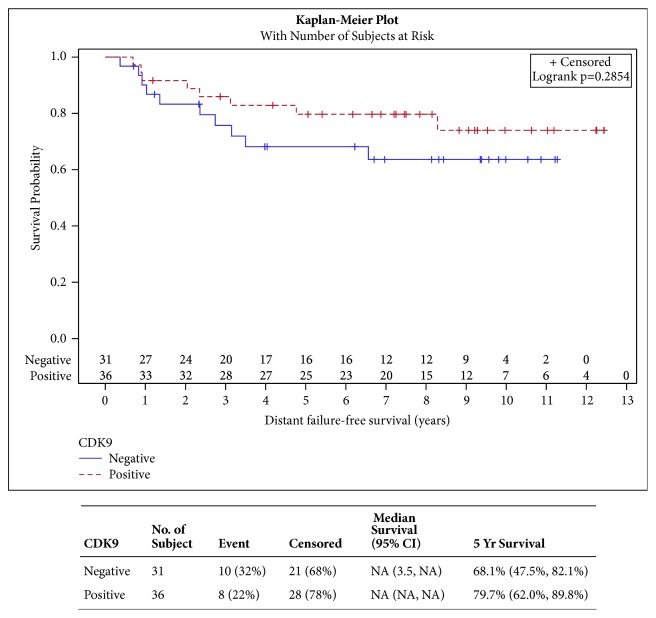
**Distant failure-free survival**. Figure 2 shows distant failure-free survival curves for patients with CDK9 positive versus CDK9 negative tumors. This shows a statistically nonsignificant difference in the two survival curves. However, there is a trend for increased distant failure free survival in the group of patients with positive CDK9.

**Figure 3:**
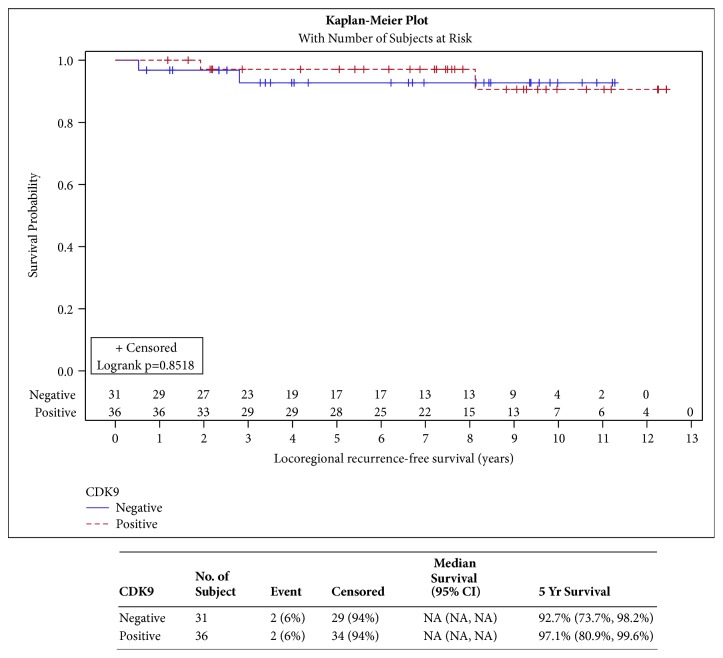
**Locoregional recurrence-free survival**. Figure 3 illustrates locoregional recurrence-free survival curves for patients with CDK9 positive versus CDK9 negative tumors. There is no statistically significant or observable difference in the two survival curves.

**Figure 4:**
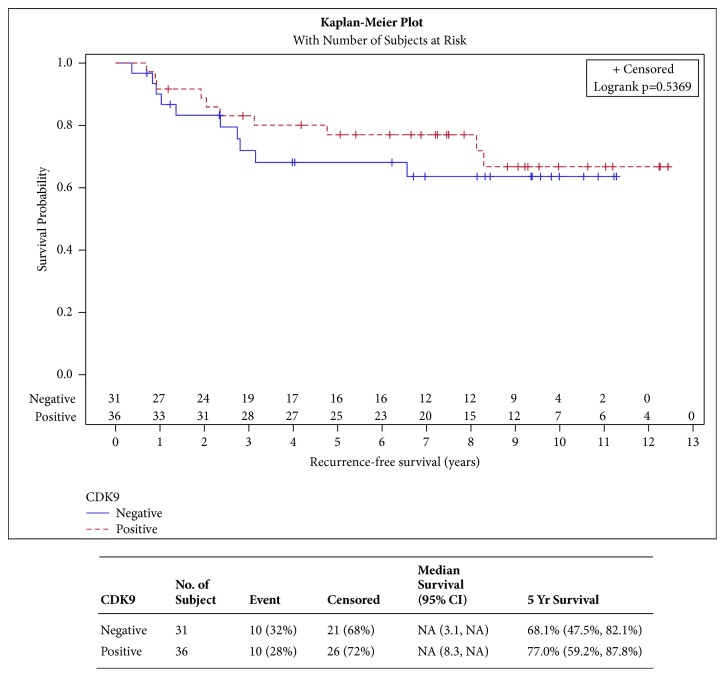
**Recurrence-free survival**. Figure 4 shows curves comparing recurrence-free survival in patients with CDK9 positive versus CDK9 negative tumors. There is no statistically significant or observable difference in the two survival curves.

**Figure 5:**
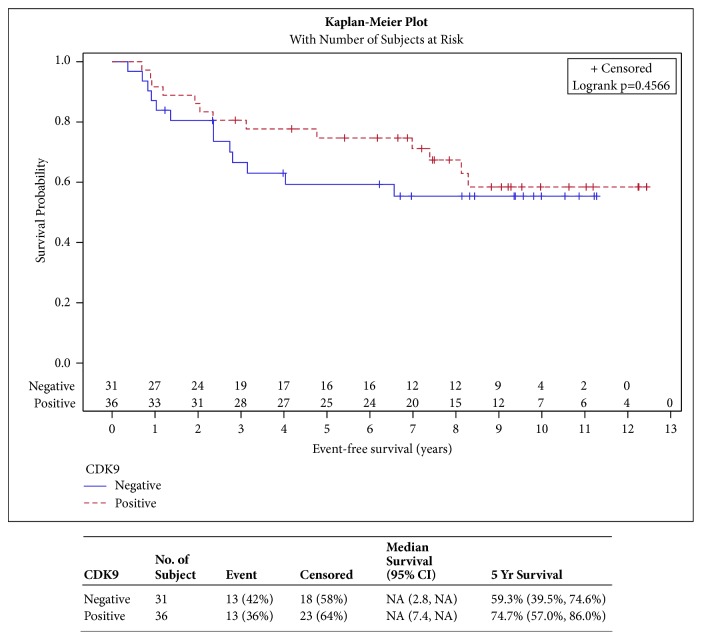
**Event-free survival**. Figure 5 shows curves comparing event-free survival in patients with CDK9 positive versus CDK9 negative tumors. There is no statistically significant or observable difference in the two survival curves.

CDK9 expression was not shown to have a significant impact on DFFS, LRR, RFS, and EFS (Figure 2, p=0.2854, Figure 3 p=0.8518, Figure 4 p=0.5369, and Figure 5 p=0.4566, respectively). CDK9 expression was not significantly associated with age, stage, receptor status, or breast cancer subtype (Table 3).

## 4. Discussion

Our study aimed to determine if CDK9 expression in residual breast tumors following the use of NACT correlated with clinical outcomes. We found that high CDK9 expression was significantly associated with improved OS beyond 3 years, suggesting that high CDK9 expression is a favorable prognostic factor for long-term clinical outcome. We also observed a trend towards improved DFFS but not LRFS with high CDK9 expression compared with low CDK9 expression, suggesting that CDK9 expression may be more prognostic for distant failure. Interestingly, we did not observe any significant separation of the OS curve before 3 years, suggesting that CDK9 expression is not a good surrogate for short-term response to treatment. Given that low CDK9 expression leads to increased spontaneous DNA damage and genetic instability, we speculate that patients with residual breast tumors with low CDK9 expression may have more aggressive disease and thus poorer prognoses. This paradoxical observation is in contrast to another study investigating CDK9 expression in patients with de novo pancreatic adenocarcinoma tumors, which showed that high CDK9 expression has worse clinical outcome [21] as well as its current investigation as a therapeutic target for cancer treatment [22–25].

In addition, while high expression of CKD9 was observed in fewer TNBC then ER+/PR+/Her2neu- tumors, this was not statistically significant. Due to low event numbers, a multivariable analysis was unable to be performed. Future studies using an increased sample size could yield stronger and more statistically significant results.

One of the difficulties with the study was loss of tumor core samples during the TMA staining process. During staining, some samples either became detached from the TMA slide or folded over themselves. Those particular samples became unreadable by the pathologist and had to be excluded from analysis. This resulted in significant decrease of the study sample size. Future studies using the TMA staining process should attempt to place multiple core samples per patient. The probability of losing multiple samples is lower and would allow for more patient data to be analyzed. In addition, the number of events available for analysis was small. Although a multivariate analysis is desirable, this was not feasible given the low number of events for each time point. A longer follow-up period may be required to obtain a proper sample of events and demonstrate significance in our data. Increasing the patient cohort number could also provide the additional data necessary to reach statistical significance. Ultimately an independent cohort, as well as a randomized clinical trial, may validate the prognostic significance of CDK9 expression following neoadjuvant chemotherapy. One potential benefit of this work is that, with further validation, patients with low CDK9 expression in residual breast tumors following NACT may benefit from more aggressive adjuvant therapies, including enrollment in clinical trials of novel therapies.

## 5. Conclusions

The results of this study do not show a significant relationship between CDK9 expression in residual breast tumors following NACT and improved patient LRR, RFS, and EFS. However, our findings of improved survival after 3 years suggest that, with further validation, CDK9 expression could have a future role as a prognostic biomarker for patients with breast cancer who fail to achieve a pathologic complete response after NACT.

## Figures and Tables

**Table 1:** **Descriptive Statistics**. This table shows clinicopathologic characteristics of the study participants including demographic information, tumor characteristics, and survival data.

**Variable**	**Level**	**N = 84**	%
CDK9	Negative	31	46.3
	Positive	36	53.7
	Missing	17	-

ER status	Negative	32	39.0
	Positive	50	61.0
	Missing	2	-

PR status	Negative	40	48.8
	Positive	42	51.2
	Missing	2	-

Her2Neu status	Negative	63	77.8
	Positive	18	22.2
	Missing	3	-

Subtype	Triple negative	22	27.5
	ER+/PR+/Her2Neu-	33	41.3
	Other	25	31.3
	Missing	4	-

Age at diagnosis (years)	<50	32	38.1
	50+	52	61.9

Stage	2	43	52.4
	3	39	47.6
	Missing	2	-

Death	No	53	63.1
	Yes	31	36.9

Locoregional recurrence	No	78	92.9
	Yes	6	7.1

Distant failure	No	61	72.6
	Yes	23	27.4

Recurrence (LR or Distant)	No	58	69.0
	Yes	26	31.0

Event (Recurrence or Death)	No	49	58.3
	Yes	35	41.7

**Table 2:** **Overall survival-3 years**. This table shows an increase in overall survival after 3 years with tumors positive for CDK9 as compared to those negative for CDK9.

		**Overall survival (years)**		
**Covariate**	**Level**	**Hazard Ratio**	**HR P-value**	**Type3 P-value**
CDK9 (Less than 3 years)	Positive	0.99 (0.30-3.23)	0.982	0.982
	Negative	-		

CDK9 (Greater than 3 years)	Positive	0.26 (0.07-0.98)	**0.046**	**0.046**
	Negative	-		

Number of observations in the original data set = 84.

Number of observations used = 67.

**Table 3:** **CDK9 expression and breast cancer subtype/receptor status**. This tables shows no significant association of CDK9 expression with breast cancer subtype and receptor status.

			**CDK9**		
**Covariate**	**Statistics**	**Level**	**Negative N=31**	**Positive N=36**	**P-value** **∗**
ER status	N (Col %)	Negative	14 (46.67)	9 (25)	0.066
	N (Col %)	Positive	16 (53.33)	27 (75)	

PR status	N (Col %)	Negative	17 (56.67)	12 (33.33)	0.057
	N (Col %)	Positive	13 (43.33)	24 (66.67)	

Her2Neu status	N (Col %)	Negative	23 (76.67)	30 (83.33)	0.498
	N (Col %)	Positive	7 (23.33)	6 (16.67)	

Subtype	N (Col %)	Triple negative	10 (34.48)	7 (19.44)	0.088
	N (Col %)	ER+/PR+/Her2Neu-	9 (31.03)	21 (58.33)	
	N (Col %)	Other	10 (34.48)	8 (22.22)	

Age at diagnosis (years)	N (Col %)	<50	11 (35.48)	14 (38.89)	0.774
	N (Col %)	50+	20 (64.52)	22 (61.11)	

Stage	N (Col %)	2	16 (53.33)	20 (57.14)	0.758
	N (Col %)	3	14 (46.67)	15 (42.86)	

*∗*The p-value is calculated by chi-square test or Fisher's exact, where appropriate.

## Data Availability

The data used to support the findings of this study are available from the corresponding author upon request.
